# Photoinduced homolytic C–H activation in *N*-(4-homoadamantyl)phthalimide

**DOI:** 10.3762/bjoc.7.36

**Published:** 2011-03-02

**Authors:** Nikola Cindro, Margareta Horvat, Kata Mlinarić-Majerski, Axel G Griesbeck, Nikola Basarić

**Affiliations:** 1 Department of Organic Chemistry and Biochemistry, Ruđer Bošković Institute, Bijenička cesta 54, 10 000 Zagreb, Croatia; 2 Department of Chemistry, University of Cologne, Greinstr. 4, Cologne D-50939, Germany

**Keywords:** homoadamantanes, photoinduced H-abstraction, phthalimides

## Abstract

*N*-(4-homoadamantyl)phthalimide (**5**) on excitation and population of the triplet excited state underwent intramolecular H-abstractions and gave products **6** and **7**. The major product, *exo*-alcohol **6** was a result of the regioselective δ H-abstraction and the stereoselective cyclization of the 1,5-biradical. Minor products **7** were formed by photoinduced γ H-abstractions, followed by ring closure to azetidinols and ring enlargement to azepinediones. The observed selectivity to *exo*-alcohol **6** was explained by the conformation of **5** and the best orientation and the availability of the δ-H for the abstraction.

## Introduction

Since the pioneering work of Ciamican and Paterno [1–2], the photochemistry of ketones has been intensively studied [3–6]. One important chemical pathway for the deactivation of ketones from the electronically excited states is photoinduced H-abstraction [7]. Intermolecular photoinduced H-abstraction leads principally to the reduction of the ketone [8–12], whereas intramolecular H-abstraction [13–14] leads to cyclization [15–17] or fragmentation, the so called Norish type II reaction [18–20]. The photochemistry of phthalimide derivatives is often similar to that of simple ketones [21–33]. For example, phthalimide derivatives in the electronically excited state abstract H-atoms from alcohols to give reduction products [34]. Furthermore, suitably substituted phthalimides deactivate from the excited state by intramolecular H-abstractions to yield cyclization products, often benzazepinone derivatives [35–37]. Therefore, photoinduced homolytic C–H activation by phthalimide derivatives can, in principle, be used in organic synthesis for the preparation of benzazepinones [21,25,33].

**Scheme 1:**
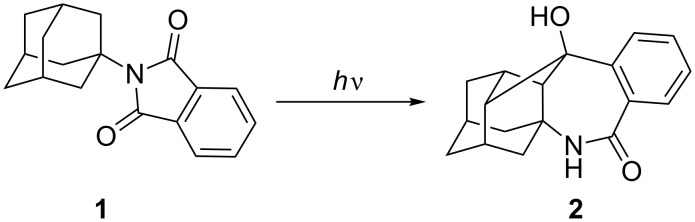
Photoinduced domino reaction of adamantylphthalimide.

In continuation of our interest in the Majerski's laboratory in the functionalization and transformation of cage molecules [38–48] as well as the preparation of biologically active compounds [49], we turned our attention to adamantylphthalimides [50–52]. Recently, in cooperation with the group of Griesbeck we discovered a photoinduced domino reaction of adamantylphthalimide that involves two consecutive γ H-abstractions and leads to a complex methanoadamantane benzazepinone **2** (Scheme 1) [51]. The mechanism of the photoinduced domino reaction was investigated and it was found that it probably takes place from a higher excited triplet state or the singlet state [52]. Herein, we report the synthesis and photochemistry of a phthalimide derivative of homoadamantane **5**. The research was conducted to investigate the availability of different C–H bonds in the homoadamantane skeleton for the homolytic activation, that is, abstraction by the phthalimide. The research was, furthermore, sparked by the discovery that numerous poly-azaheterocyclic adamantane derivatives show antiviral activity [53–55]. Thus, photoproducts derived from **5** may also exhibit antiviral activity, although that is yet to be substantiated.

## Results and Discussion

**Scheme 2:**
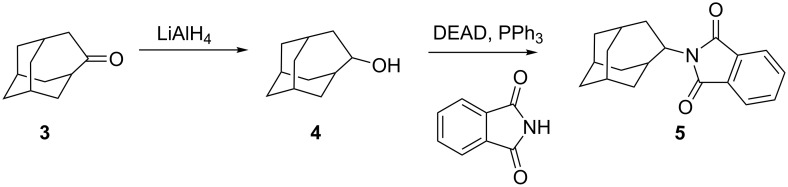
Synthesis of homoadamantylphthalimide **5**.

The synthesis of homoadamantylphthalimide **5** started from homoadamantanone **3** which is easily prepared by the ring enlargement of adamantanone with diazomethane, generated in situ from *N*-methyl-*N*-nitroso-*p*-toluenesulfonamide (Diazald^®^) [56]. Homoadamantanone **3** was first reduced to the alcohol **4** [57–59] and subsequently converted to homoadamantyl phthalimide **5** in moderate yield via the Mitsunobu protocol [60] (Scheme 2).

**Figure 1:**
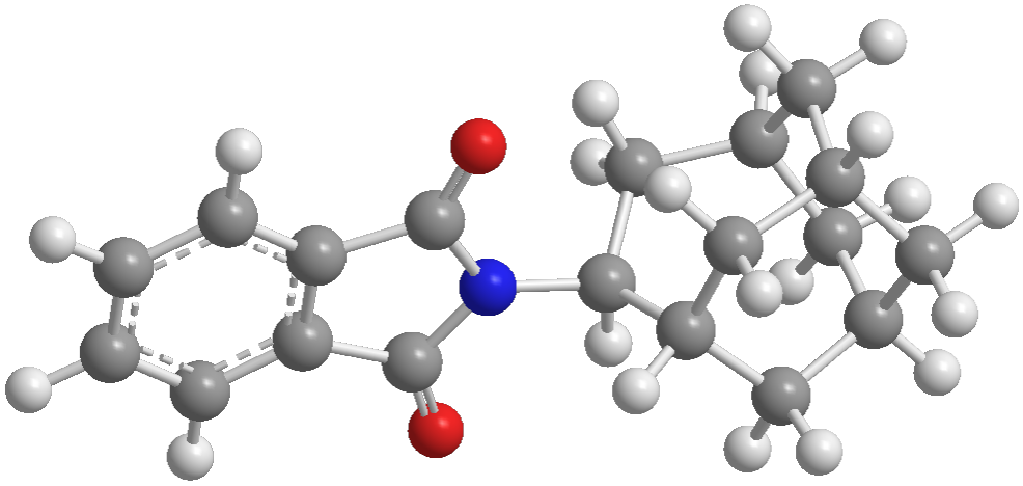
Molecular structure of **5**, the geometry optimization was performed by use of DFT B3LYP/6-31G.

To get more insight into the availability of H-atoms in the homoadamantane skeleton for the abstraction by the phthalimide, molecular modeling was performed. The geometry of **5** was optimized by DFT B3LYP/6-31G method (Figure 1) [61]. Investigation of the distances between the carbonyl groups of the phthalimide moiety and the H-atoms of the homoadamantane skeleton revealed that there are principally two γ H-atoms (with respect to the carbonyl of the phthalimide) and one δ H-atom available for the abstraction (see Scheme 4 and the Experimental for the notation of atoms). The distances between the carbonyl and γ H-atoms at the positions 3 and 5 of the homoadamantane skeleton are 3.52 and 2.52 Å, respectively, whereas distance to the δ H-atom at the position 2 is 2.37 Å. The calculated distances suggest that phthalimide **5** in the excited state should primarily give rise to products derived from the abstraction of the γ H-atom from position 5 and the δ H-atom from position 2 in the homoadamantane skeleton.

**Scheme 3:**
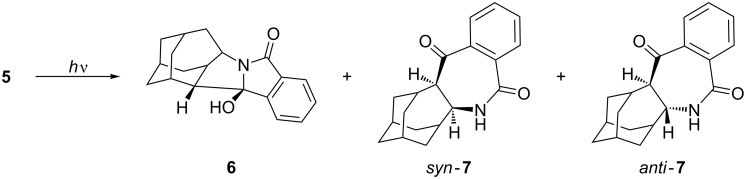
Products after irradiation of **5**.

Irradiation of **5** was performed in CH_3_CN, CH_3_CN–H_2_O (3:1), acetone and acetone–H_2_O (3:1) and gave three products, i.e., the *exo*-alcohol **6**, and the azepinediones *syn*-**7** and *anti*-**7** (Scheme 3). In all investigated solvents, *exo*-alcohol **6** was the major product. For example, when the irradiation was performed in acetone–H_2_O for 4 h (see the Experimental), the ratio of the isolated starting phthalimide **5** and the photoproducts **6** and **7** was 10:8:6. However, prolonged irradiation in acetone–H_2_O (18 h) gave only alcohol **6** which was isolated in 53% yield. Products **7** decomposed on prolonged irradiation. The photochemical reaction was more efficient in solvent system containing acetone rather than CH_3_CN. After 18 h irradiation in acetone–H_2_O, complete conversion of **5** was achieved, whereas under the same photolysis conditions in CH_3_CN–H_2_O, 72% of unreacted **5** was recovered. This finding is in accordance with acetone acting as a triplet sensitizer and the anticipated triplet state reactivity of the phthalimide in the H-abstraction reactions. Furthermore, the addition of H_2_O as a protic solvent also increased the reactivity of the phthalimide, based on the conversion of the starting material under the same irradiation conditions. Thus, after 1 h photolysis of **5** in acetone, only 5% of **5** was converted to products, whereas after photolysis in acetone–H_2_O, a 60% conversion was achieved. This suggests that phthalimide in the triplet excited state, in a protic solvent, undergoes H-abstraction to give products with ten times higher quantum yields than in an aprotic solvent. Such a finding is in accordance with previous reports for phthalimides and is probably due to a switching of the relative order of the singlet and the triplet excited states of the phthalimide [54,62–63].

The structures of photoproducts was determined by spectroscopic methods. In the ^1^H NMR spectrum of **6**, in the aromatic region, 4 well-resolved signals are present, indicating the loss of symmetry of the aromatic part of the molecule (compared to the symmetry in **5**). Similarly, in the aromatic part of the ^13^C NMR spectrum, 4 doublets are present. In the aliphatic part of the ^13^C spectrum, 6 doublets and 5 triplets are observed and one quaternary C at δ 98.13 ppm. All of these features are in agreement with the molecular structure of **6**. The structure is further supported by 2D NMR wherein all the observed interactions are in agreement with the structure (for HMBC see the Experimental). The *exo*-stereochemistry of the product was established from the NOESY spectrum where an NOE interaction between the H-7 and H-10, and the H-7 and H-15 were present (for the notation of atoms see the Experimental). Assignment of the structure to azepinone products **7** was straightforward from the corresponding NMR spectra. In the ^1^H NMR spectra they display the characteristic broad N–H singlets at 6.4 and 6.0 ppm. Furthermore, in the aliphatic part of the ^13^C NMR spectra, 6 doublets and 5 triplets are present, in accord with the proposed structures. However, from their spectra we were unable to assign the *syn*- or the *anti*-stereochemistry to the isolated products **7**. In the NOESY spectra of both isolated compounds, an NOE interaction was observed between the H-atoms at the position 1 and 11 (for the notation of atoms see the Experimental), which precluded unambiguous assignment of the stereochemistry to the isomers **7**.

**Scheme 4:**
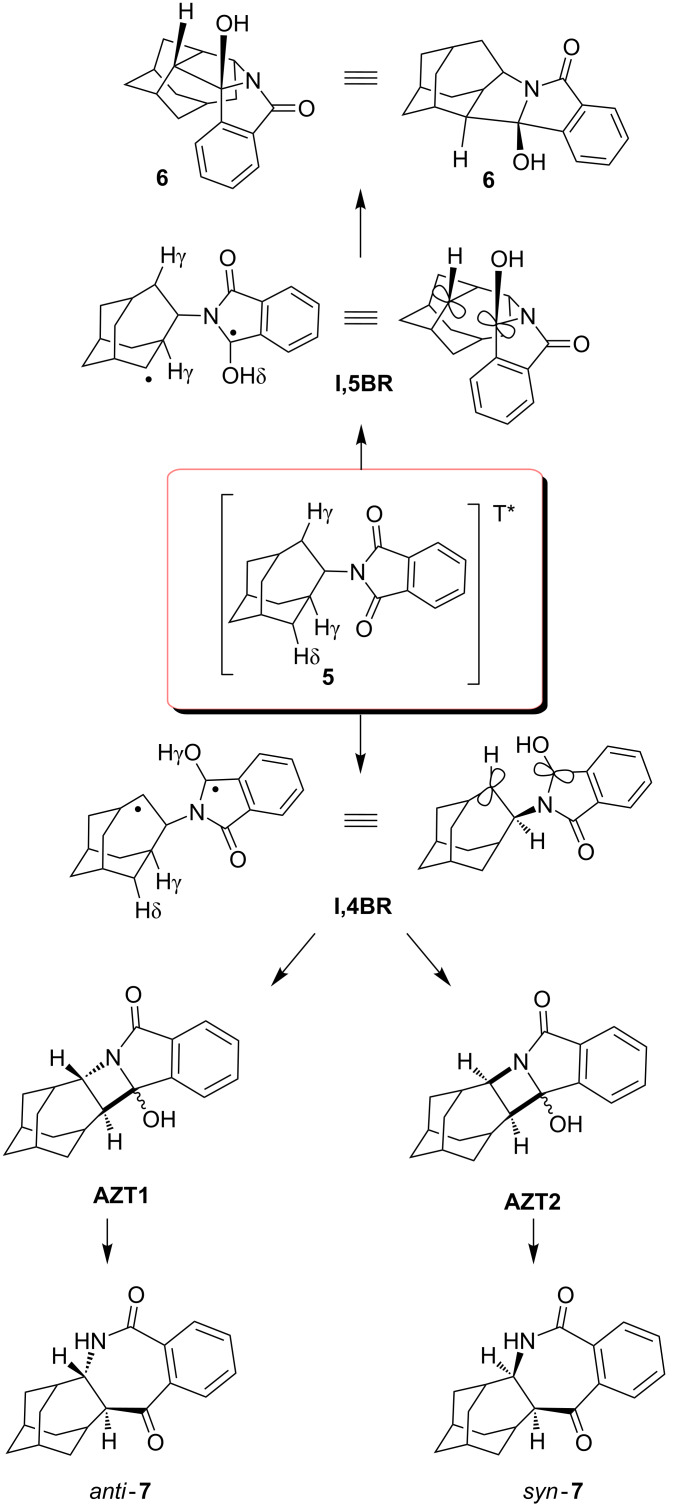
Proposed mechanism for the photochemical transformation of **5**.

According to the above product study, a mechanism for the photochemical transformation of **5** can be proposed. On excitation (direct or sensitized) the triplet state of **5** is populated and undergoes intramolecular H-abstraction. The abstraction of the δ H-atom of the homoadamantane (Scheme 4) is probably the fastest since this H-atom is closest to the carbonyl of the phthalimide moiety. Although two γ H-atoms are available for the abstraction, and it is generally known that γ H-atoms are more readily abstracted [7,13–14], the conformation of the molecule is probably the most important factor that directs the selective δ-abstraction. The δ-abstraction gives rise to a 1,5-biradical (**1,5BR**) that undergoes stereoselective cyclization to furnish the major product, *exo*-alcohol **6**. The observed selectivity of the 1,5-cyclization is in line with the stability of the product formed. According to B3LYP calculations [61], *exo*-**6** is 15 kJ/mol more stable than *endo*-**6**. However, the reason for the observed selectivity is probably due to the preferred motion in which the half-filled orbitals of **1,5BR** overlap after ISC, and induce ring-closure.

Two γ H-atoms in **5** are in the proximity to the carbonyl and, in principle, available for abstraction. However, the H-atom at the ethylene bridge of the homoadamantane skeleton (position 5) is closer to the carbonyl, and therefore, more readily abstracted. Abstraction gives a 1,4-biradical (**1,4BR**) that cyclizes to azetidinol intermediates **AZT1** and **AZT2**. Azetidinols undergo subsequent ring enlargement to furnish products *anti*-**7** and *syn*-**7**, respectively. The ratio of the isolated compounds **7** is 5:1. However, no assignment of their stereochemistry was made from their spectra. Nevertheless, the cyclization of **1,4BR** is probably selective giving more **AZT2**, and product *syn*-**7** from the subsequent ring enlargement. The reason for the suspected selectivity becomes evident from the inspection of the structure of **1,4BR** where ring closure to the azetidinol probably takes place preferably from the upper side giving **AZT2**. The other approach (rear side) is sterically more demanding and results in a less stable trans-configuration on the azetidinol intermediate **AZT1**. Consequently, we suspect that the major isomer **7** has the *syn*-configuration, whereas the minor isomer has the *anti*-configuration.

## Conclusion

*N*-(4-homoadamantyl)phthalimide (**5**) was synthesized and its photochemistry investigated. On excitation and population of the triplet state, **5** undergoes intramolecular homolytic C–H activation and gives products **6** and **7**. The major product of the photochemical reaction is exo-alcohol **6** formed *via* regioselective δ H-abstraction and stereoselective cyclization of the 1,5-biradical. The observed selectivity is due to the conformation of **5** where the δ H-atom is the closest to the carbonyl of the phthalimide moiety. Minor products **7** are formed by photoinduced γ H-abstraction, followed by ring closure to azetidinols and ring enlargement to azepinediones. High selectivity and high isolable yield of **6** in the photoreaction of **5** makes this photoinduced C–H activation useful in the synthesis of very complex derivatives with the homoadamantane skeleton with potential antiviral activity for which otherwise tedious multi-step synthesis would be required.

## Experimental

### General

^1^H and ^13^C NMR spectra were recorded on a Bruker Spectrometer at 300 or 600 MHz. All NMR spectra were measured in CDCl_3_ using tetramethylsilane as a reference. High-resolution mass spectra (HRMS) were measured on an Applied Biosystems 4800 Plus MALDI TOF/TOF instrument. Melting points were obtained using an Original Kofler apparatus and are uncorrected. IR spectra were recorded on Perkin Elmer M-297 and ABB Bomem M-102 spectrophotometers. Solvents were purified by distillation. 4-Homoadamantanone (**3**) was prepared in the laboratory according to a known procedure [56]. Phthalimide, triphenylphosphine, lithium aluminum hydride (LAH) and diethyl azodicarboxylate (DEAD) were obtained from commercial sources. All photochemical experiments were performed in a Rayonet photochemical reactor equipped with 300 nm lamps.

#### 4-Homoadamantanol (**4**)

To a suspension of LAH (3.00 g, 78.95 mmol) in dry THF (100 mL), was added a solution of 4-homoadamantanone (**3**, 1.00 g, 6.08 mmol) in THF (50 mL). The suspension was heated under reflux for 24 h. After the reaction was complete and cooled (ice bath), LAH was carefully destroyed by the slow addition of H_2_O. The resulting precipitate was removed by filtration and washed with diethyl ether (3 × 30 mL). The combined organic solution was dried over anhydrous MgSO_4_. After filtration and removal of the solvent under vacuum, compound **4** [57–59] was isolated in 99% yield (998 mg) and used in the next step without further purification.

#### *N*-(4-homoadamantyl)phthalimide (**5**)

4-Homoadamantanol (**4**, 1.00 g, 6.06 mmol), phthalimide (1.23 g, 8.35 mmol) and DEAD (2.00 mL, 12.64 mmol) were dissolved in dry THF (60 mL) in a three necked round bottomed flask. To the resulting mixture, a solution of triphenylphosphine (2.00 g, 7.63 mmol) in THF (60 mL) was added over a 1 h period. The reaction mixture was stirred at rt in the dark and under a nitrogen atmosphere for 12 h. After the reaction was complete, the solvent was evaporated, and the crude product purified by column chromatography on silica gel with hexane–CH_2_Cl_2_ (1:1) as eluent. Pure **5** (622 mg, 35%) was obtained as a crystalline product.


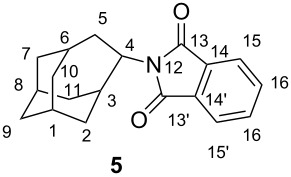


Colorless crystals, mp 110–111 °C; ^1^H NMR (CDCl_3_, 300 MHz, δ/ppm) 7.84–7.77 (m, 2H), 7.72–7.66 (m, 2H), 4.61 (t, 1H, *J* = 9.5 Hz, H-4), 2.60–2.50 (m, 1H), 2.45 (d, 1H, *J* = 14.1 Hz), 2.22–2.03 (m, 3 H), 1.98–1.85 (m, 5H), 1.80–1.62 (m, 3H), 1.60–1.52 (m, 3H); ^13^C NMR (CDCl_3_, 75 MHz, δ/ppm) 170.00 (s, 2C, C-13 and C-13'), 133.60 (d, 2C, C-15 and C-15' or C-16 and C-16'), 131.96 (s, 2C, C-14 and C-14'), 122.82 (d, 2C, C-15 and C-15' or C-16 and C-16') 56.97 (d, C-4), 40.10 (t, C-2 or C-10), 39.74 (t, C-2 or C-10), 39.56 (d, C-3), 36.64 (t, C-5), 35.58 (t, C-11), 33.99 (t, C-7 or C-9), 31.53 (t, C-7 or C-9), 29.44 (d, C-1), 27.18 (d, C-6), 27.17 (d, C-8); IR (KBr) ν/cm^−1^ 2910, 2850, 1765, 1707, 1375, 1350, 1323, 1114, 1084, 1070, 710; HRMS (MALDI), calculated for C_19_H_22_NO_2_ 296.1645, observed 296.1644.

#### General procedure for semi-preparative photolysis of *N*-(4-homoadamantyl)phthalimide (**5**)

Phthalimide **5** (10 mg, 0.034 mmol) was dissolved in 20 mL of the appropriate solvent, CH_3_CN, CH_3_CN–H_2_O (3:1), acetone or acetone–H_2_O (3:1) in a quartz cuvette. The solutions were purged with N_2_ for 20 min and irradiated in a Rayonet at 300 nm for 1 h. The solvent was removed on a rotary evaporator and ^1^H NMR spectrum of the crude photolysis mixture recorded to determine the ratio of the products.

#### Preparative photolysis of *N*-(4-homoadamantyl)phthalimide (**5**)

A Pyrex vessel was filled with a solution of *N*-(4-homoadamantyl)phthalimide (**5**) (100 mg, 0.338 mmol) in acetone–H_2_O (3:1, 200 mL). The solution was irradiated in a Rayonet photoreactor at 300 nm for 4 h. During irradiation the reaction mixture was continuously purged with argon and cooled with a finger-condenser with tap water. After irradiation, the solvent was removed on a rotary evaporator. Unreacted **5** (42%) was recovered by column chromatography on silica gel with 5% MeOH-CH_2_Cl_2_ as eluent. The photoproducts were isolated by repeated preparative thin layer chromatography with the use of the following solvent mixtures: 5% MeOH-CH_2_Cl_2_, 30% diethyl ether-CH_2_Cl_2_, ethyl acetate–diethyl ether–CH_2_Cl_2_ (1:1:3) and hexane–ethyl acetate–diethyl ether–CH_2_Cl_2_ (1:2:2:5).

#### 1-aza-9-hydroxyhexacyclo[10.7.0.1^13,17^.0^3,8^.0^10,15^.0^11,19^]eicosa-3,5,7-trien-2-one (**6**)


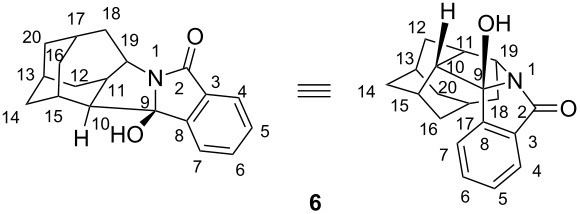


33 mg (33%); Colorless crystals; mp 183–185 °C; ^1^H NMR (CDCl_3_, 300 MHz, δ/ppm) 7.71 (td, 1H, *J* = 1.0 7.5 Hz, H-4), 7.61 (dt, 1H, *J* = 1.0, 7.5 Hz, H-6), 7.54 (td, 1H, *J* = 1.0, 7.5 Hz, H-7), 7.46 (dt, 1H, *J* = 1.0, 7.5 Hz, H-5), 4.35 (t(dd), 1H, *J* = 7.3 Hz, H-19), 2.70 (t(dd), 1H, *J* = 5.8 Hz, H-10), 2.60 (d, 1H, *J* = 14 Hz, H-16), 2.38 (br s, 1H, H-15), 2.11–2.22 (m, 3H, 2H-18, H-13), 2.03 (q, 1H, *J* = 6.9 Hz, H-11), 1.88–1.98 (m, 2H, H-17 and H-20), 1.70–1.88 (m, 3H, H-16, H-14 and H-12), 1.55–1.62 (m, 3H, H-14 and H-12), 1.43 (d, *J* = 13 Hz, H-20); ^13^C NMR (CDCl_3_, 75 MHz, δ/ppm) 177.27 (s, C-2), 151.77 (s, C-8), 133.65 (d, C-6), 131.38 (s, C-3), 129.52 (d, C-5), 123.95 (d, C-4), 122.24 (d, C-7), 98.13 (s, C-9), 64.31 (d, C-19), 48.19 (d, C-10), 42.21 (d, C-11), 40.94 (t, C-18), 40.38 (t, C-20), 35.62 (t, C-14), 32.69 (t, C-16), 31.17 (d, C-13), 30.62 (t, C-12), 27.93 (d, C-15), 26.89 (d, C-17); IR (KBr) ν/cm^−1^ 3300, 2980, 2902, 1694, 1605, 1464, 1433, 1338, 1320, 1297, 1231, 1125, 1053; HRMS (MALDI), calculated for C_19_H_22_NO_2_ 296.1645, observed 296.1649.

Important HMBC interactions: H-19 and C-6, C-2, C-9, C-10; H-17 and C-19; H-16 and C-14, C-20; H-15 and C-9; H-12 and C-19; H-10 and C-19, C-8; H-4 and C-2, C-8; H-6 and C-8; H-7 and C-9; Important NOE interactions: H-7 and H-15; H-17 and H-10.

#### *rel*-(1*R*,11*S*)-2-azapentacyclo[9.7.0.1^12,16^.1^14,18^.0^4,9^]eicosa-4,6,8-trien-3,10-dione (*syn*-**7**) and *rel*-(1*S*,11*S*)-2-azapentacyclo[9.7.0.1^12,16^.1^14,18^.0^4,9^]eicosa-4,6,8-trien-3,10-dione (*anti*-**7**)


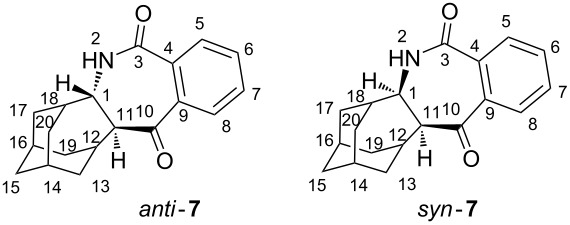


21 mg (21%); colorless crystals, mp 244–245 °C; ^1^H NMR (CDCl_3_, 300 MHz, δ/ppm) 7.90–7.85 (m, 1H), 7.62–7.54 (m, 2H), 7.33–3.28 (m, 1H), 6.02 (br s, 1H, NH), 4.43 (m, H-1), 3.09 (d, 1H, *J* = 8.0 Hz, H-11), 3.04 (m, 1H, H-12), 2.44 (m (ddd), 1H, H-18), 2.18 (s, 1H, H-13), 2.08–1.93 (m, 4H, H-14, H-15, H-19 and H-17 or H-20), 1.89–1.83 (m, 2H, H-17 and H-20), 1.75 (d, 1H, H-13), 1.68–1.57 (m, 3H, 2 H-15 and H-20 or H-17), 1.38 (d, *J* = 12 Hz, H-19); ^13^C NMR (CDCl_3_, 75 MHz, δ/ppm) 132.30 (d, 1C), 130.38 (d, 1C), 128.71 (d, 1C), 125.40 (d, 1C), 67.21 (d, C-11), 54.32 (d, C-1), 41.42 (t, C-19), 35.50 (t, C-15), 34.72 (d, C-18), 32.75 (t, C-17 or C-20), 32.69 (t, C-17 or C-20), 30.78 (t, C-13), 28.61 (d, C-12), 27.09 (d, C-16 or C-14), 27.05 (d, C-16 or C-14), quarternary C-signals were not detected; IR (KBr) ν/cm^−1^ 3424, 2922, 2856, 1703, 1658, 1561, 1384, 1275, 1096, 801; HRMS (MALDI), calculated for C_19_H_22_NO_2_ 296.1645, observed 296.1646.

Important COSY interactions: NH and H-1, H-1 and H-11, H-1 and H-18. Important NOE interaction: H-1 and H-11, H-1 and H-18, NH and H-17, H-11 and H-19 [64].

4 mg (4%); colorless crystals, mp 229–231 °C; ^1^H NMR (CDCl_3_, 300 MHz, δ/ppm) 8.12 (dd, 1H, *J* = 1.2, 7.6 Hz, H-5 or H-8), 8.06 (dd, 1H, *J* = 1.2, 7.6 Hz , H-5 or H-8), 7.72–7.60 (m, 2H, H-6 and H-7), 6.49 (br s, 1H, NH), 3.92 (t, 1H, *J* = 7.5 Hz, H-1), 3.00 (t, 1H, *J* = 5.4 Hz, H-12), 2.95 (d, 1H, *J* = 8.8 Hz, H-11), 2.17–1.82 (m, 7H, H-18, 2 H-13, H-14, H-17, H-16, H-19), 1.63–1.40 (m, 6H, 2 H-15, H-17, 2 H-20, H-19); ^13^C NMR (CDCl_3_, 75 MHz, δ/ppm) 133.01 (d, C-5 or C-8), 131.66 (d, C-5 or C-8), 130.81 (d, C-6 or C-7), 129.48 (d, C-6 or C-17), 67.33 (d, C-11), 58.49 (d, C-1), 40.43 (t, C-17 or C-19), 38.73 (t, C-17 or C-19), 36.00 (d, C-18), 35.78 (t, C-20), 32.06 (t, C-15), 30.36 (d, C-12), 30.18 (t, C-13), 26.48 (d, C-14 or C-16), 26.17 (d, C-14 or C-16), quarternary C-signals were not detected due to small quantity of the sample; IR (KBr) ν/cm^−1^ 3159, 3057, 2899, 2851, 1681, 1658, 1595, 1441, 1403, 1275, 1266, 1011, 786, 756; HRMS (MALDI), calculated for C_19_H_22_NO_2_ 296.1645, observed 296.1646. Important NOE interaction: H-1 and H-11, H-1 and H-18, NH and H-20 [64].

## Supporting Information

File 1 Supporting information contains ^1^H and ^13^ C NMR spectra of compounds **5**–**7** and atomic coordinates for **5** and **6** calculated by B3LYP/6-31G.
