# Supplementary material for: Photoinduced homolytic C–H activation in N-(4-homoadamantyl)phthalimide
Source: Beilstein J Org Chem. 2011 Mar 2;7:270–7. doi: 10.3762/bjoc.7.36 (PMC3062987; doi:10.3762/bjoc.7.36)
Supplement: File 1 — Supporting information contains 1H and 13 C NMR spectra of compounds 5–7 and atomic coordinates for 5 and 6 calculated by B3LYP/6-31G. [file Beilstein_J_Org_Chem-07-270-s001.pdf]

Supporting Information

for

Photoinduced homolytic C–H activation in *N*-(4-homoadamantyl)phthalimide

Nikola Cindro[1], Margareta Horvat[1], Kata Mlinarić-Majerski[1], Axel G. Griesbeck[2], and Nikola Basarić[1]*

Address: [1]Department of Organic Chemistry and Biochemistry, Ruđer Bošković Institute, Bijenička cesta 54, 10 000 Zagreb, Croatia and [2] Department of Chemistry, University of Cologne, Greinstr. 4, Cologne D-50939, Germany

Email: Nikola Basarić - nbasaric@irb.hr

* Corresponding author

^{1}H and ^{13}C NMR spectra of compounds 5–7 and atomic coordinates for 5 and 6 calculated by B3LYP/6-31G.

Table of contents:

1. Atomic coordinates for **5** and **6** obtained by B3LYP/6-31G S3

2. ^{1}H NMR ($CDCl_3$, 300 MHz) of **5** S8

3. ^{13}C NMR ($CDCl_3$, 75 MHz) of **5** S9

4. ^{1}H NMR ($CDCl_3$, 300 MHz) of **6** S10

5. ^{13}C NMR ($CDCl_3$, 75 MHz) of **6** S11

6. ^{1}H NMR ($CDCl_3$, 300 MHz) of *syn*- or *anti*-**7**... S12

7. ^{13}C NMR ($CDCl_3$, 75 MHz) of *syn*- or *anti*-**7**.. S13

8. ^{1}H NMR ($CDCl_3$, 300 MHz) of *anti*- or *syn*-**7**... S14

9. ^{13}C NMR ($CDCl_3$, 75 MHz) of *anti*- or *syn*-**7**.. S15

Atomic coordinates for 5 and 6 obtained by B3LYP/6-31G

Atomic coordinates for **5**

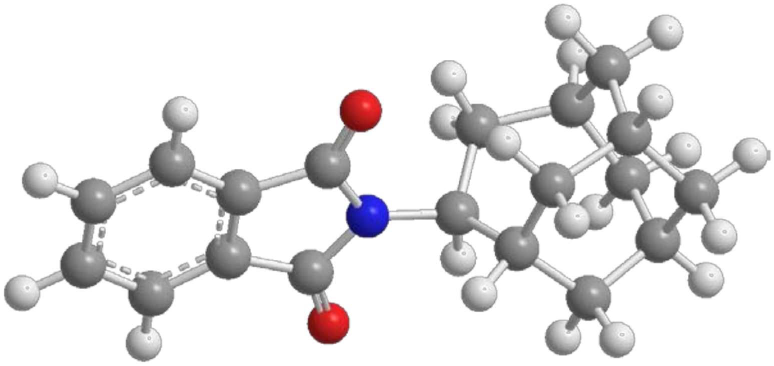

Center Number	Atomic Number	Atomic Type	Coordinates (Angstroms) X	Y	Z
1	6	0	3.747463	1.094034	-0.458456
2	6	0	3.499793	1.088388	1.077092
3	6	0	2.718577	-0.144053	1.624397
4	6	0	1.382019	0.283712	-1.094483
5	6	0	2.481720	1.359036	-1.301601
6	6	0	4.343976	-0.258886	-0.905583
7	6	0	3.239127	-1.458121	0.988447
8	6	0	1.979760	-1.145740	-1.253608
9	6	0	3.344025	-1.380454	-0.548604
10	6	0	1.174141	-0.036278	1.528890
11	6	0	0.610878	0.581825	0.228864
12	7	0	-0.842691	0.295385	0.120686
13	6	0	-1.802335	1.337830	0.101991
14	6	0	-3.132725	0.680153	0.012864
15	6	0	-2.929707	-0.706264	-0.014436
16	6	0	-1.464203	-0.967817	0.056630
17	6	0	-4.407723	1.228048	-0.039073
18	6	0	-5.493322	0.338546	-0.121718
19	6	0	-5.289783	-1.050005	-0.150305
20	6	0	-3.994346	-1.593687	-0.096263
21	8	0	-0.892957	-2.070338	0.062065
22	8	0	-1.539333	2.547797	0.155451
23	1	0	4.467881	1.897316	-0.670822
24	1	0	2.994269	2.016876	1.380059
25	1	0	4.484577	1.102225	1.566358
26	1	0	2.937259	-0.201733	2.700963
27	1	0	0.641783	0.417571	-1.896046
28	1	0	2.774404	1.364886	-2.361409
29	1	0	2.072736	2.355495	-1.085021

30	1	0	5.306249	-0.442966	-0.407018
31	1	0	4.538451	-0.240570	-1.987143
32	1	0	2.588171	-2.290428	1.287154
33	1	0	4.239875	-1.678395	1.389596
34	1	0	2.133969	-1.310082	-2.330569
35	1	0	1.252139	-1.892940	-0.921119
36	1	0	3.735179	-2.342482	-0.910421
37	1	0	0.800374	0.571630	2.364205
38	1	0	0.758096	-1.040849	1.659685
39	1	0	0.621395	1.671215	0.339320
40	1	0	-4.555645	2.301933	-0.016272
41	1	0	-6.504248	0.730003	-0.164428
42	1	0	-6.146285	-1.712845	-0.214886
43	1	0	-3.827488	-2.664821	-0.117372

Atomic coordinate for *endo*-**6**

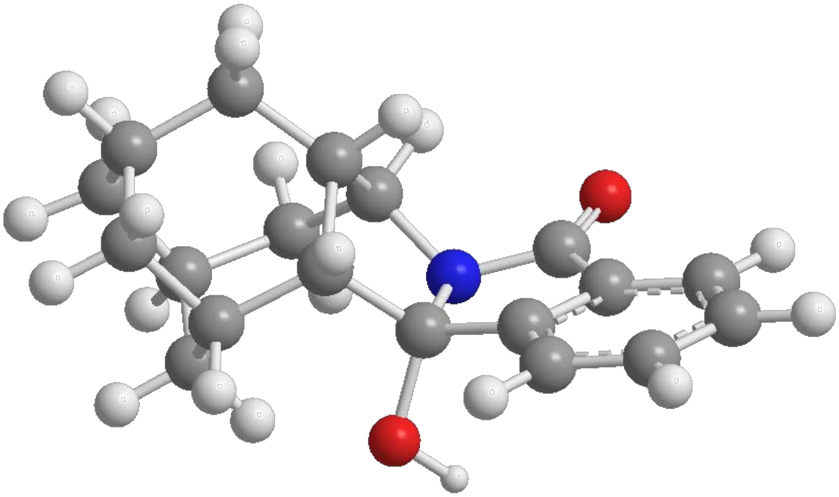

Center Number	Atomic Number	Atomic Type	Coordinates (Angstroms) X	Y	Z
1	6	0	3.349788	-0.558642	-1.228903
2	6	0	2.838826	-1.864934	-0.577283
3	6	0	1.736862	-1.530688	0.457847
4	6	0	2.346569	-0.613073	1.550448

5	6	0	2.981554	0.688303	1.011474
6	6	0	3.954387	0.368933	-0.152617
7	6	0	2.197546	0.109104	-2.010383
8	6	0	0.512468	-0.967768	-0.324104
9	6	0	0.887668	0.259805	-1.213548
10	6	0	0.749769	1.465298	-0.236518
11	6	0	1.950768	1.797468	0.678323
12	7	0	-0.500005	1.061143	0.484013
13	6	0	-0.647985	-0.420850	0.556072
14	6	0	-2.093100	-0.628925	0.114571
15	6	0	-2.723517	0.613969	-0.023738
16	6	0	-1.707305	1.688293	0.127632
17	8	0	-1.844610	2.901777	-0.109472
18	6	0	-2.817939	-1.810076	-0.026244
19	6	0	-4.180936	-1.715878	-0.349775
20	6	0	-4.805625	-0.466205	-0.501735
21	6	0	-4.077875	0.719110	-0.334494
22	8	0	-0.513783	-0.958261	1.901241
23	1	0	4.143458	-0.804304	-1.949317
24	1	0	3.667732	-2.384377	-0.077667
25	1	0	2.451752	-2.545430	-1.349816
26	1	0	1.407161	-2.450648	0.955534
27	1	0	3.135020	-1.197524	2.049100
28	1	0	1.591986	-0.386195	2.304886
29	1	0	3.593832	1.109173	1.824058
30	1	0	4.303350	1.303067	-0.615947
31	1	0	4.843599	-0.125488	0.266338
32	1	0	1.982448	-0.506050	-2.896094
33	1	0	2.512735	1.091799	-2.388923
34	1	0	0.134087	-1.783029	-0.954141
35	1	0	0.076003	0.391341	-1.945544
36	1	0	0.487038	2.376738	-0.783446
37	1	0	2.500386	2.631309	0.218369
38	1	0	1.533628	2.186817	1.615180
39	1	0	-2.346309	-2.777754	0.109735
40	1	0	-4.762630	-2.622501	-0.483376
41	1	0	-5.860890	-0.422257	-0.750449
42	1	0	-4.541530	1.693188	-0.447068
43	1	0	-1.197214	-0.553463	2.474195

Atomic coordinate for *exo*-**6**

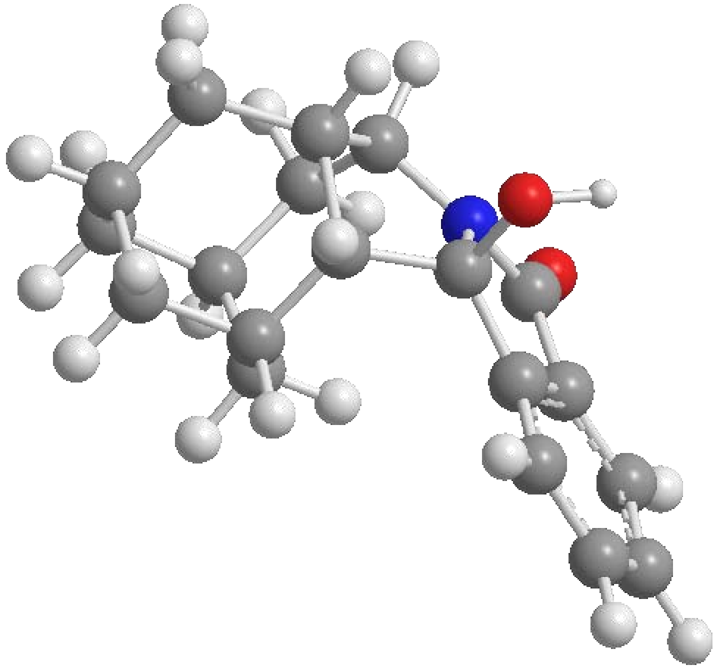

Center Number	Atomic Number	Atomic Type	Coordinates (Angstroms) X	Y	Z
1	6	0	-3.451006	-0.789176	-0.416477
2	6	0	-2.462010	-1.866623	-0.919785
3	6	0	-1.015633	-1.321727	-0.823166
4	6	0	-0.901268	-0.036168	-1.692249
5	6	0	-1.951020	1.067393	-1.380884
6	6	0	-3.367758	0.443066	-1.339726
7	6	0	-3.161820	-0.455383	1.069294
8	6	0	-0.688558	-1.175776	0.682783
9	6	0	-1.676979	-0.208031	1.412756
10	6	0	-1.092391	1.214733	1.121837
11	6	0	-1.650169	1.932309	-0.125688
12	7	0	0.360901	0.912928	0.990061
13	6	0	0.656591	-0.530149	1.049658
14	6	0	1.932631	-0.636893	0.236283
15	6	0	2.375429	0.657253	-0.082277
16	6	0	1.354096	1.652293	0.364702
17	8	0	1.360930	2.884723	0.169111
18	6	0	2.704661	-1.748893	-0.087699
19	6	0	3.911647	-1.539949	-0.775726
20	6	0	4.342317	-0.244936	-1.106335
21	6	0	3.574140	0.874429	-0.755326
22	8	0	0.906864	-0.987532	2.421010

23	1	0	-4.473512	-1.189668	-0.472545
24	1	0	-2.696178	-2.128567	-1.960481
25	1	0	-2.560525	-2.785686	-0.324964
26	1	0	-0.321878	-2.062632	-1.245799
27	1	0	-1.055572	-0.352563	-2.734806
28	1	0	0.111636	0.376255	-1.653986
29	1	0	-1.933654	1.765780	-2.230630
30	1	0	-4.097744	1.202415	-1.026614
31	1	0	-3.651641	0.133965	-2.356916
32	1	0	-3.499113	-1.307113	1.676900
33	1	0	-3.768670	0.404344	1.387030
34	1	0	-0.730077	-2.170858	1.138643
35	1	0	-1.545279	-0.382397	2.486311
36	1	0	-1.244187	1.867405	1.989202
37	1	0	-2.578991	2.438785	0.170581
38	1	0	-0.944058	2.727801	-0.386728
39	1	0	2.388703	-2.750406	0.184455
40	1	0	4.522622	-2.392892	-1.053714
41	1	0	5.280062	-0.111579	-1.635802
42	1	0	3.893024	1.882624	-0.996968
43	1	0	1.638959	-0.459677	2.803591

----

2. ^{1}H NMR (300 MHz, $CDCl_3$) of **5**

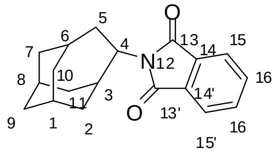

SpinWorks 2.5: NC-105

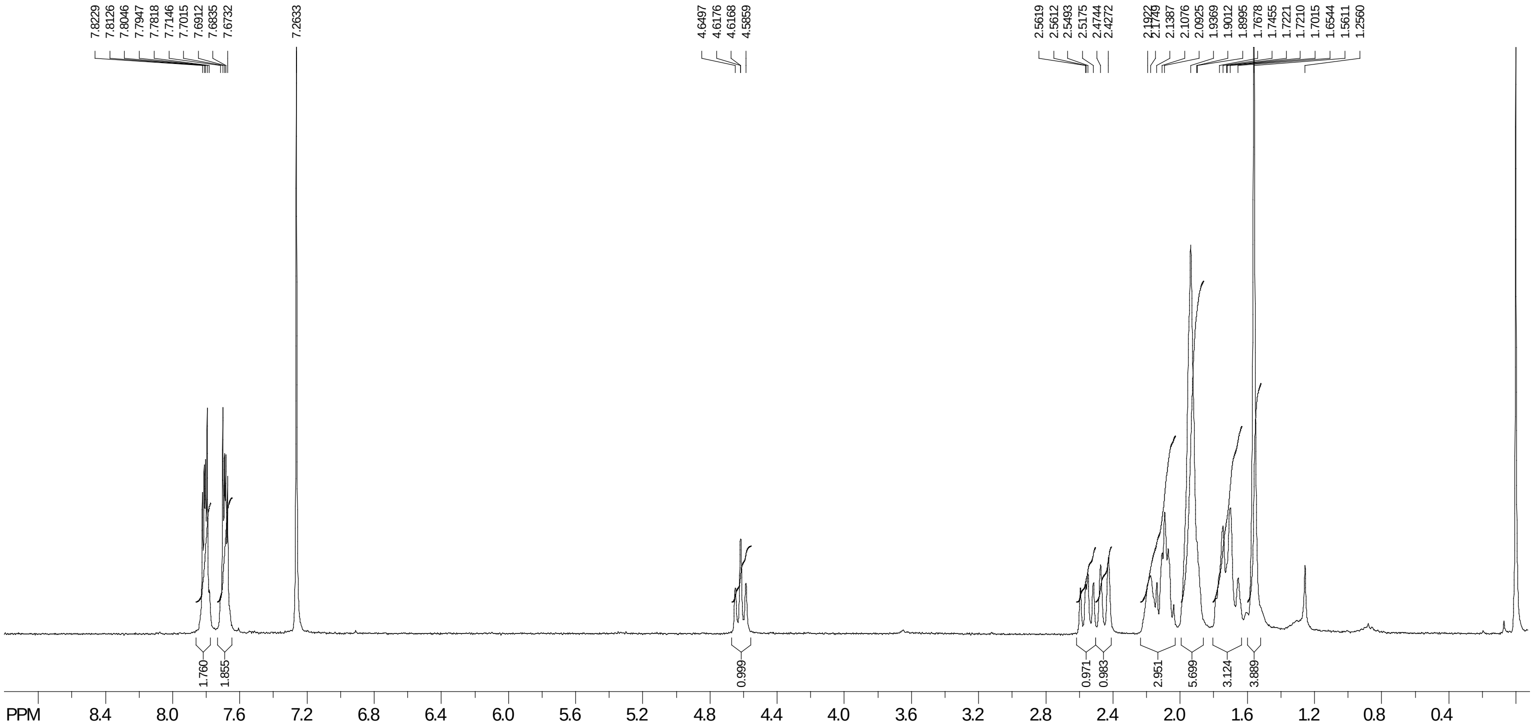

file: E:\Documents and Settings\Administrator\Desktop\nc-105\fid expt: <zg30>
transmitter freq.: 300.132701 MHz
time domain size: 32768 points
width: 6172.84 Hz = 20.567034 ppm = 0.188380 Hz/pt
number of scans: 24

freq. of 0 ppm: 300.130006 MHz
processed size: 32768 complex points
LB: 0.300 GB: 0.0000

3. ^{13}C NMR (75 MHz, $CDCl_3$) of **5**

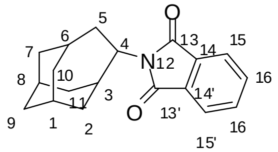

SpinWorks 2.5: NC-105-2

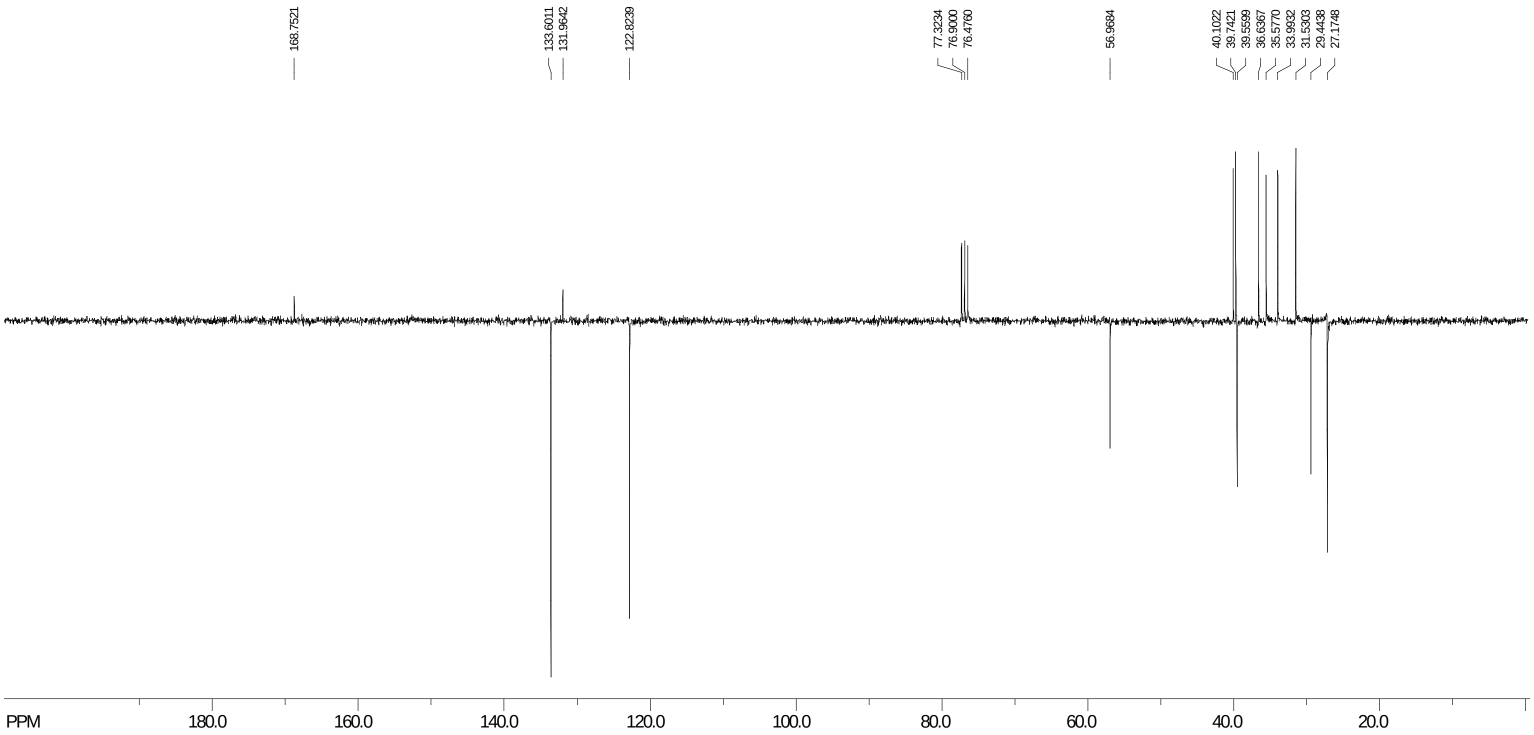

file: E:\Documents and Settings\Administrator\Desktop\nc-105-2-apt\fid expt: <jmod>
transmitter freq.: 75.475295 MHz
time domain size: 65536 points
width: 17985.61 Hz = 238.297995 ppm = 0.274439 Hz/pt
number of scans: 373

freq. of 0 ppm: 75.467758 MHz
processed size: 32768 complex points
LB: 1.000 GB: 0.0000

4. ^{1}H NMR (300 MHz, $CDCl_3$) of **6**

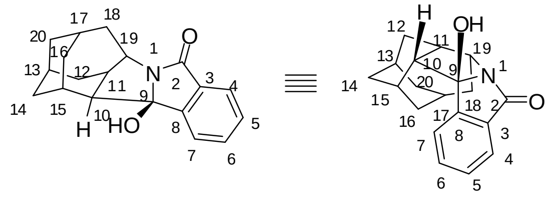

SpinWorks 2.5: NC-112-B-A

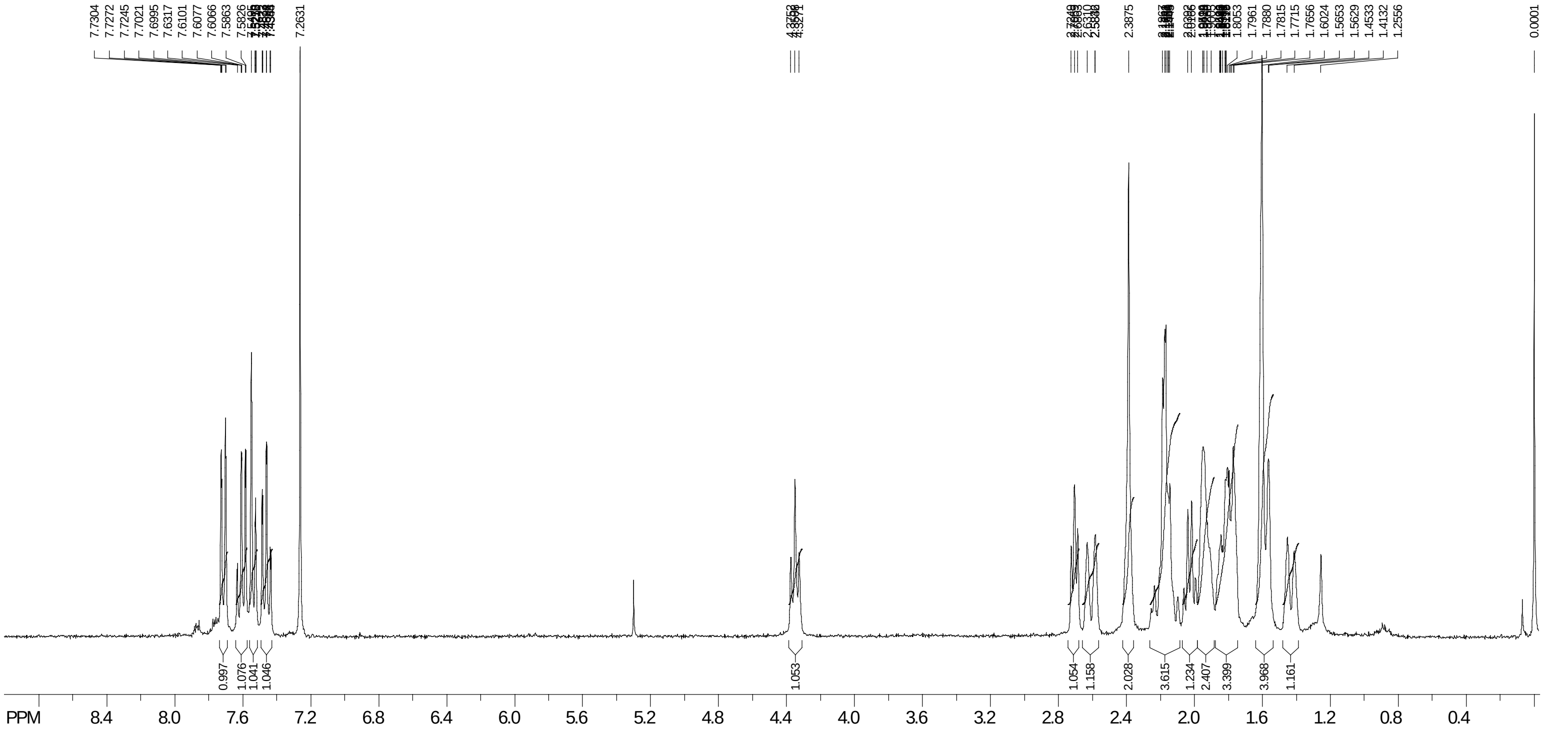

file: C:\2010\10-2010\nikola cindro\nc-112-a-b-2D\1\fid expt: <zg30>
transmitter freq.: 300.132701 MHz
time domain size: 32768 points
width: 6172.84 Hz = 20.567034 ppm = 0.188380 Hz/pt
number of scans: 24

freq. of 0 ppm: 300.130005 MHz
processed size: 32768 complex points
LB: 0.000 GB: 0.0000

5. ^{13}C NMR (75 MHz, $CDCl_3$) of **6**

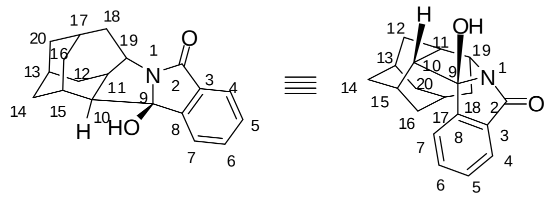

SpinWorks 2.5: NC-112-B-A

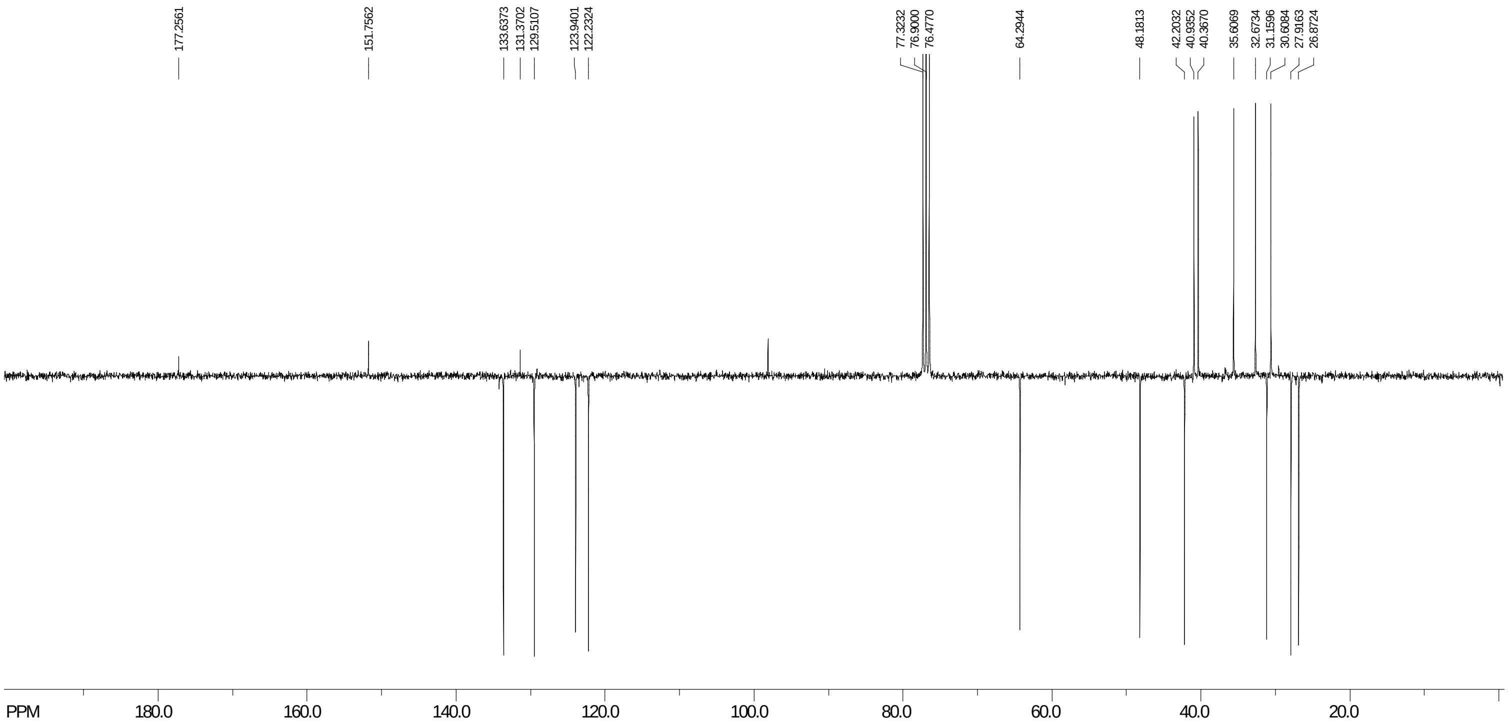

file: C:\2010\10-2010\nikola cindro\nc-112-a-b-2D\6\fid expt: <jmod>
transmitter freq.: 75.475295 MHz
time domain size: 65536 points
width: 17985.61 Hz = 238.297995 ppm = 0.274439 Hz/pt
number of scans: 16000

freq. of 0 ppm: 75.467757 MHz
processed size: 32768 complex points
LB: 1.000 GB: 0.0000

6. ^{1}H NMR (300 MHz, $CDCl_3$) of *anti*- or *syn*-**7** **(major azepinone product)**

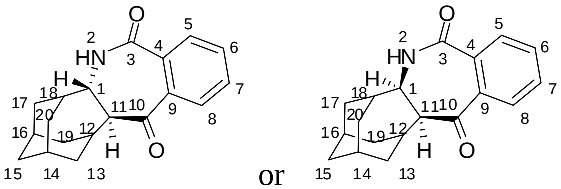

SpinWorks 2.5: NC-116-I-B-A

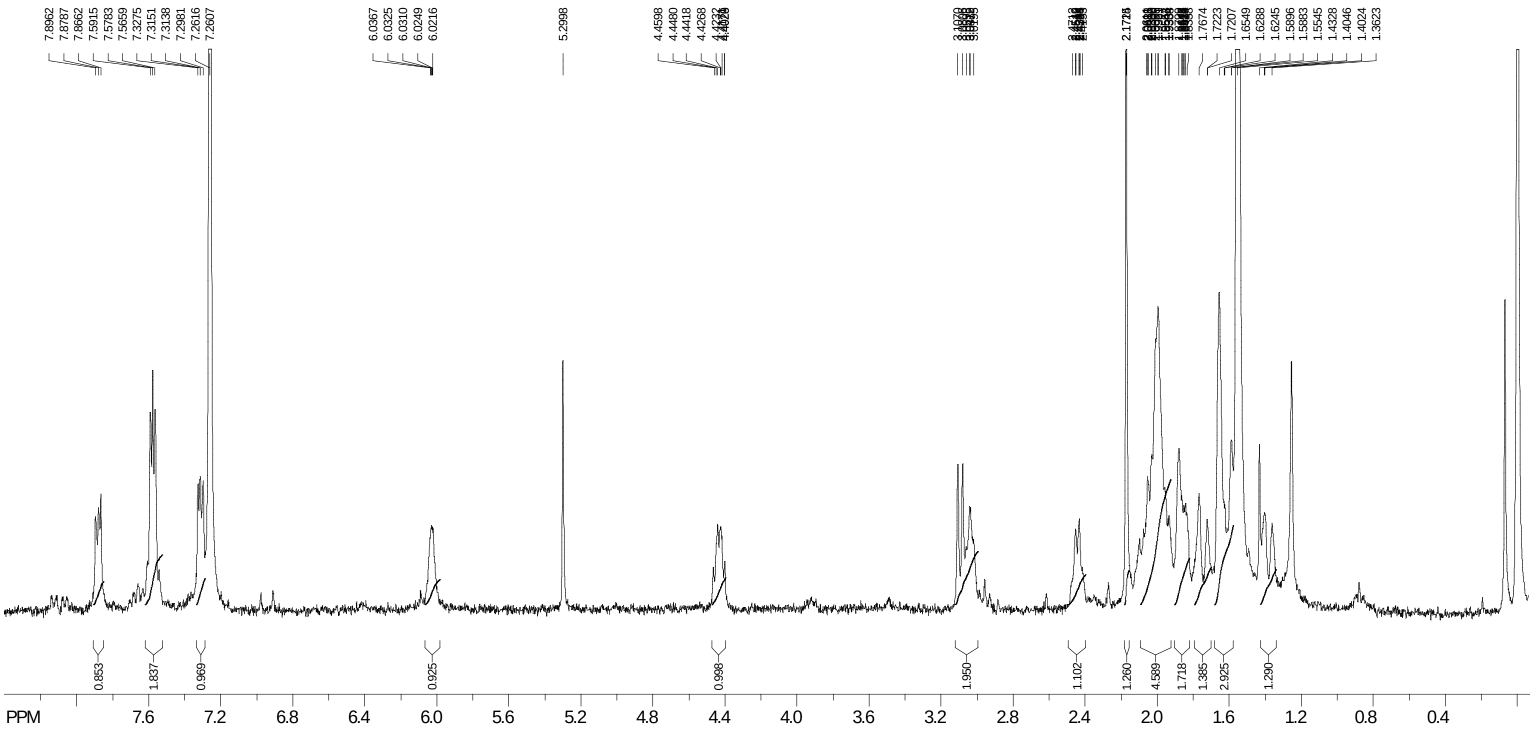

file: C:\2010\10-2010\nikola cindro\nc-116-b-a 2d\1\fid expt: <zg30>
transmitter freq.: 300.131298 MHz
time domain size: 32768 points
width: 2815.32 Hz = 9.380279 ppm = 0.085917 Hz/pt
number of scans: 47

freq. of 0 ppm: 300.130006 MHz
processed size: 32768 complex points
LB: 0.300 GB: 0.0000

7. ^{13}C NMR (75 MHz, $CDCl_3$) of *anti*- or *syn*-**7** **(major azepinone product)**

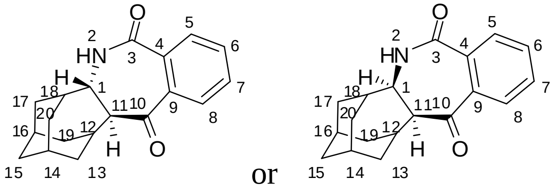

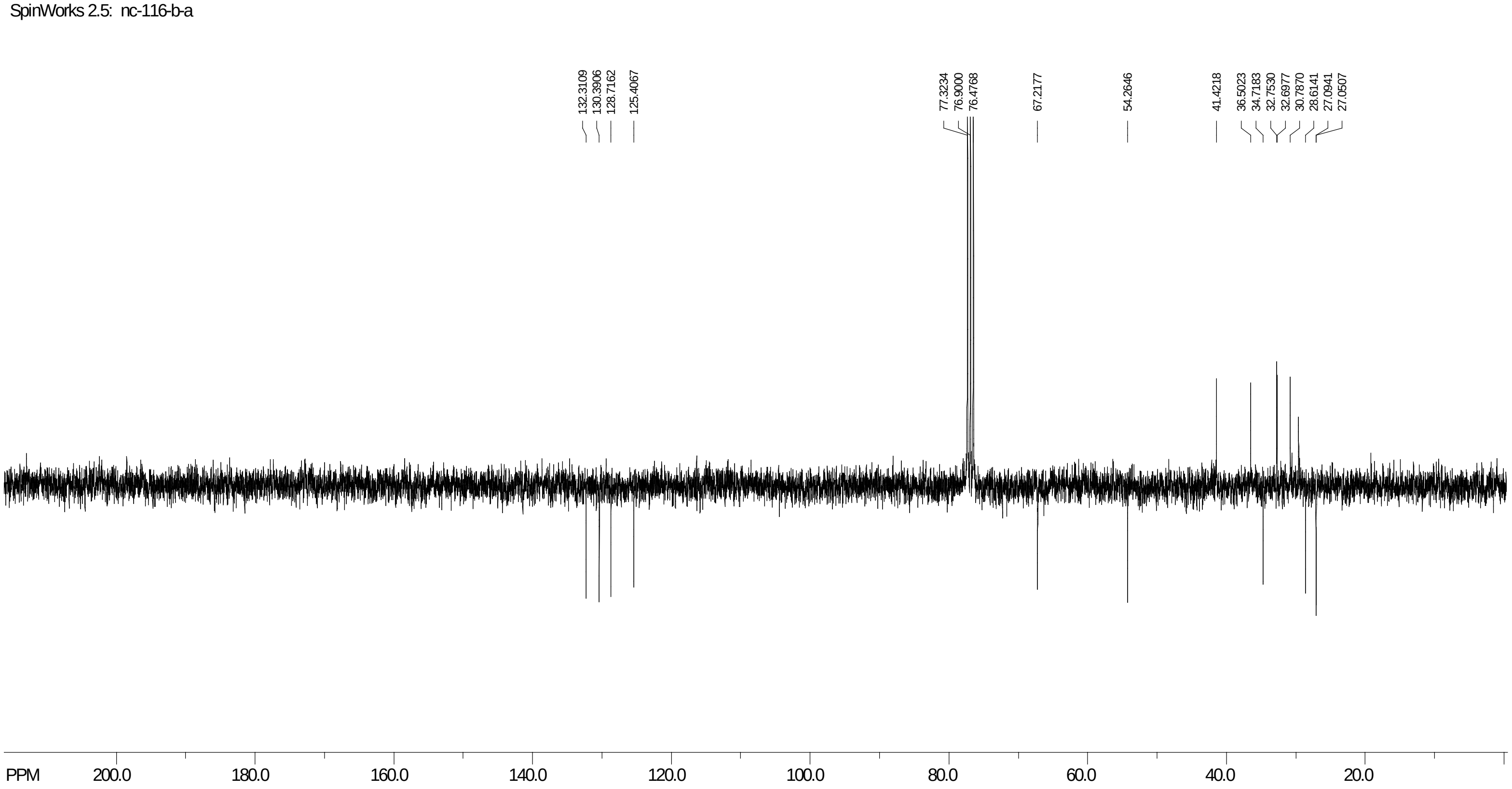

file: C:\2010\10-2010\nikola cindro\nmr-nc\nc-116-ba-apt\fid expt: <jmod>
transmitter freq.: 75.475295 MHz
time domain size: 65536 points
width: 17985.61 Hz = 238.297995 ppm = 0.274439 Hz/pt
number of scans: 2755

freq. of 0 ppm: 75.467757 MHz
processed size: 32768 complex points
LB: 0.000 GB: 0.0000

8. ^{1}H NMR (300 MHz, $CDCl_3$) of *syn*-**7** or *anti*-**7** **(minor azepinone product)**

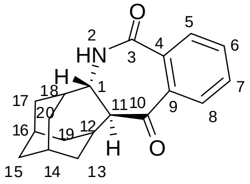

or

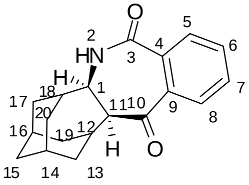

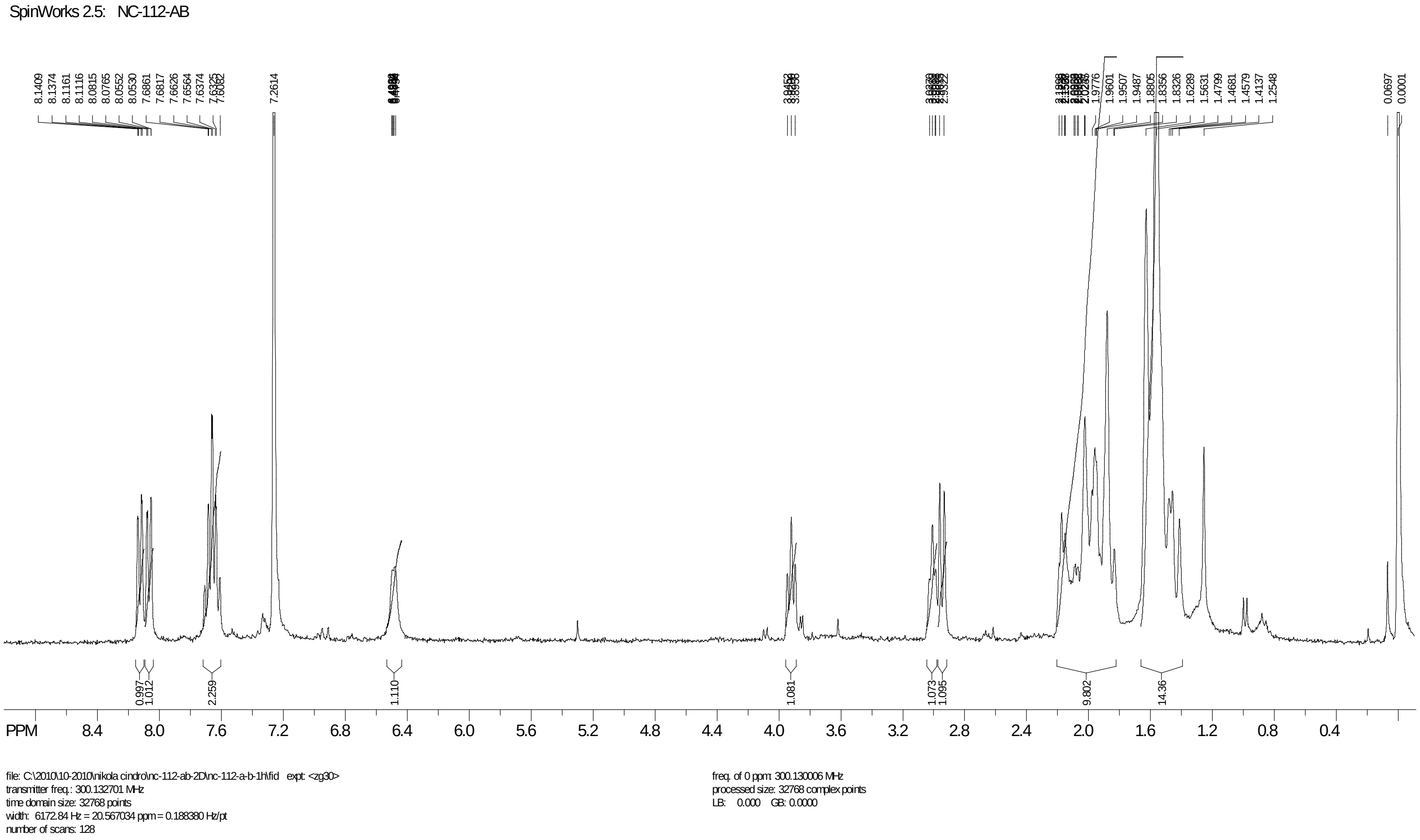

9. ^{13}C NMR (75 MHz, $CDCl_3$) of *syn*-**7** or *anti*-**7** **(minor azepinone product)**

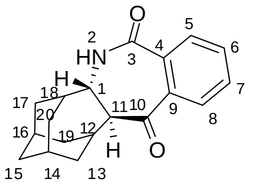

or

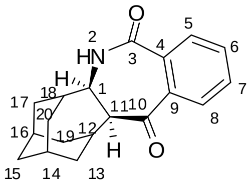

SpinWorks 2.5: NC-112-AB

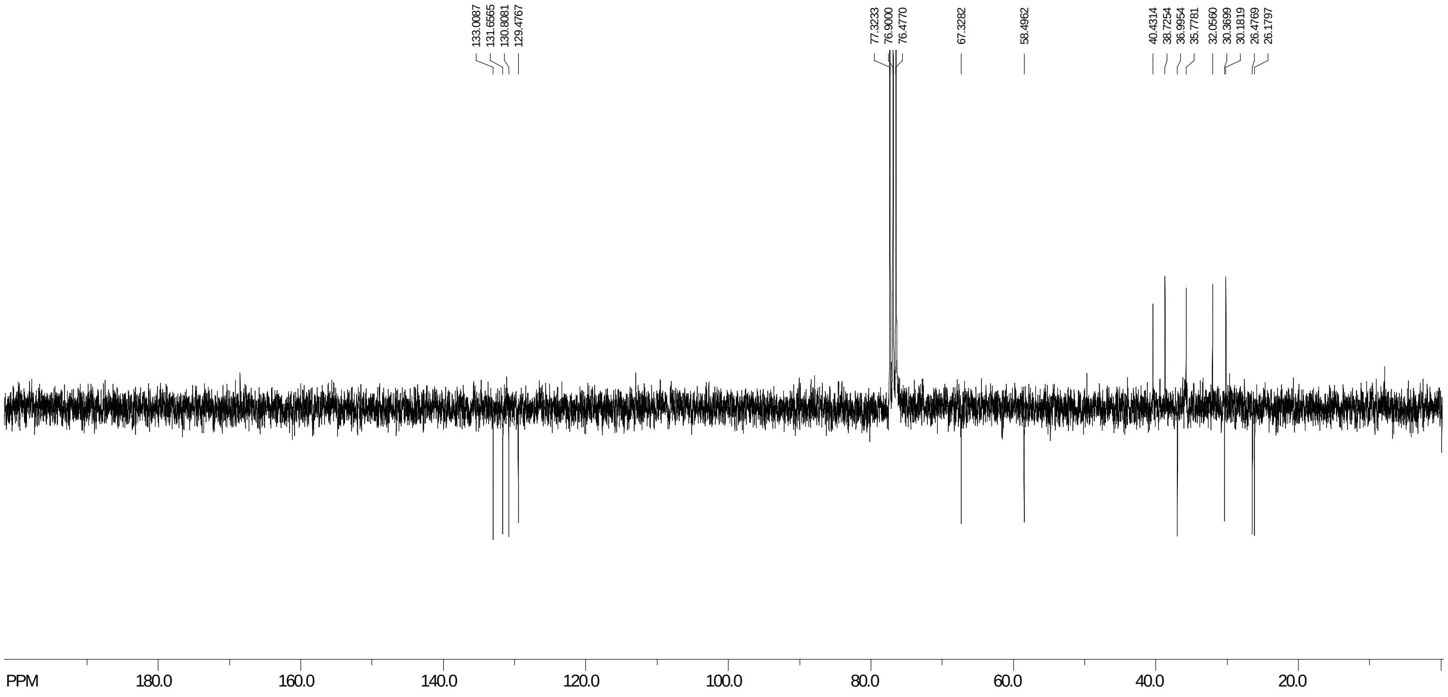

file: C:\2010\10-2010\nikola cindro\nc-112-ab-2D\nc-112-ab-apt\fid expt: <jmod>
transmitter freq.: 75.475295 MHz
time domain size: 65536 points
width: 17985.61 Hz = 238.297995 ppm = 0.274439 Hz/pt
number of scans: 6827

freq. of 0 ppm: 75.467756 MHz
processed size: 32768 complex points
LB: 0.000 GB: 0.0000
